# The Impact of the Species–Area Relationship on Estimates of Paleodiversity

**DOI:** 10.1371/journal.pbio.0030266

**Published:** 2005-07-19

**Authors:** Anthony D Barnosky, Marc A Carrasco, Edward B Davis

**Affiliations:** **1**Department of Integrative Biology and Museum of Paleontology, University of California, Berkeley, California, United States of America,; **2**Museum of Vertebrate Zoology, University of California, Berkeley, California, United States of America; Princeton UniversityUnited States of America

## Abstract

Estimates of paleodiversity patterns through time have relied on datasets that lump taxonomic occurrences from geographic areas of varying size per interval of time. In essence, such estimates assume that the species–area effect, whereby more species are recorded from larger geographic areas, is negligible for fossil data. We tested this assumption by using the newly developed Miocene Mammal Mapping Project database of western North American fossil mammals and its associated analysis tools to empirically determine the geographic area that contributed to species diversity counts in successive temporal bins. The results indicate that a species–area effect markedly influences counts of fossil species, just as variable spatial sampling influences diversity counts on the modern landscape. Removing this bias suggests some traditionally recognized peaks in paleodiversity are just artifacts of the species–area effect while others stand out as meriting further attention. This discovery means that there is great potential for refining existing time-series estimates of paleodiversity, and for using species–area relationships to more reliably understand the magnitude and timing of such biotically important events as extinction, lineage diversification, and long-term trends in ecological structure.

## Abstract 2

The importance of accounting for geographic area in estimating biodiversity from the fossil record is revealed through an analysis of the Miocene Mammal Mapping Project.

## Introduction

Paleontology-based estimates of how species diversity fluctuates through time [1–6] typically do not take into account the species–area curve, which is one of the best understood relationships in ecology and predicts how many species can be expected as the geographic area of sampling increases [7–11]. The relationship generally is expressed by *S = cA^z^,* where *S* is the number of species, *A* is the geographic area that was sampled, and *c* and *z* are empirically derived constants that express the slope of the power function and its intercept. Generally, *z*-values are lowest for accumulations of species within continental biogeographic provinces (*z* of approximately 0.15), intermediate for island archipelagos (*z* of approximately 0.25–0.45), and highest for data accumulated across continental biogeographic provinces (*z* of approximately 0.9). These relationships hold for nearly all taxonomic groups, including plants, aquatic invertebrates, insects, birds, reptiles, amphibians, and mammals [8–17], and have led to a universal recognition that the species–area relationship has high predictive power for estimating biodiversity on the modern landscape and modeling such long-term processes as extinction [8,10,11,13,18–22, but see 23,24].

Despite that recognition, it has never been possible to directly determine species–area relationships for paleontological data, which form the basis for understanding biotic processes such as how diversity fluctuates over long time periods, timing and causes of lineage diversification, and the magnitude of mass extinctions [1–6,25–29]. The importance of such assessment has been recognized [30] and therefore attempted in indirect ways, primarily by using various proxies of rock volume or outcrop area to estimate the potential for sampling taxa from various geologic time intervals [6,31,32]. Such studies have concentrated on marine invertebrate taxa and have demonstrated correlations between rock volume, outcrop area, collection effort, and paleodiversity. However, correcting for a species–area bias differs from these indirect proxies for standardization because a species–area correction must take into account the actual geographic area encompassed by the sampling sites instead of the area potentially available for sampling.

The reason that the paleospecies–area relationship has not been addressed directly stems from technological constraints and the way paleontological data were traditionally compiled, i.e., with more attention to placing them in a time interval and analyzing differences through time than to placing them in geographic context and analyzing changes across space within a given time interval. Now both of these constraints have been removed with the advent of (i) electronic paleontological databases, which include both geographic coordinates for fossil sites and lists of taxa per site [33], and (ii) Geographic Information Systems and imaging programs that allow direct measurement of the geographic area encompassed by a set of fossil species.

Here we utilize a paleontological database, Miocene Mammal Mapping Project (MIOMAP [34]), to directly derive aspects of the paleospecies–area relationship for fossil terrestrial mammals (Figure 1). The MIOMAP database is a compilation of all western North American mammal occurrences and associated geologic information between 5 and 30 million years old (details of which are fully documented online [34]). It includes approximately 3,100 georeferenced localities and 14,000 occurrences of taxa. The temporal bins are those shown in Table 1. We use this dataset because (i) we built it and are intimately familiar with its potentials and limitations; (ii) the novel mapping interfaces allow direct measurement of the area that encompasses the fossil data; (iii) the fossil localities can be well-constrained, both geographically (Figure 1) and temporally, and are well documented with metadata [34]; (iv) fossil mammals have been used for some of the most comprehensive examinations of paleodiversity fluctuations and their causes [2,4,30,35–37]; and (v) potential paleospecies–area biases have been recognized as problematic for mammals but have not been exhaustively explored [30]. Although we focus on fossil mammals, our results have implications for paleodiversity curves for any taxon.

**Figure 1:**
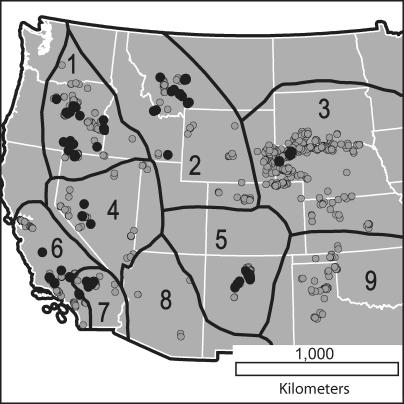
Distribution of Fossil Localities in the MIOMAP Database and Oligo-Miocene Biogeographic Provinces Gray dots show localities for all time intervals; black dots show localities that are Early Barstovian age. Black lines and numbers demarcate and label the following biogeographic provinces: 1, Columbia Plateau; 2, Northern Rockies; 3, Northern Great Plains; 4, Northern Great Basin; 5, Colorado Plateau; 6, Coastal California; 7, Mojave Desert; 8, Southern Great Basin; 9, Southern Great Plains.

**Table 1:**
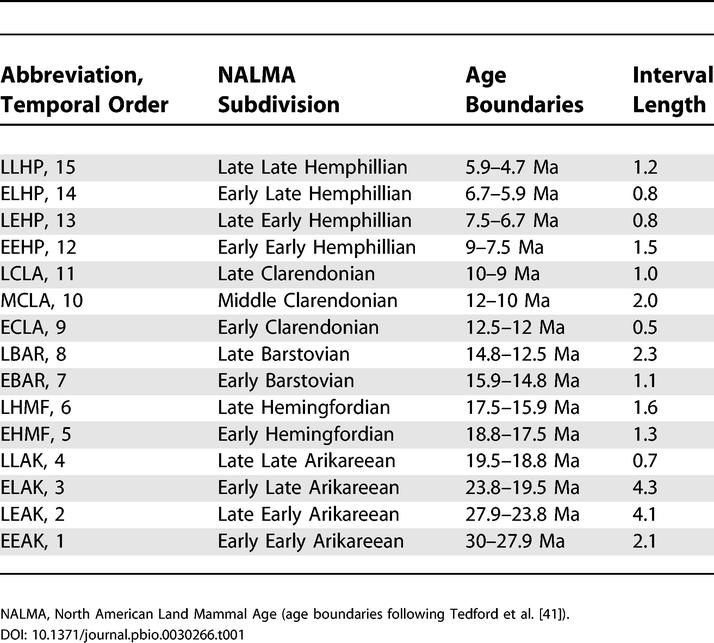
Temporal Bins into Which Species Occurrences Were Sorted

We tested for species–area effects in the fossil data in two ways. First, we derived the paleospecies–area curve for a single temporal bin for which subcontinental-scale spatial coverage was best—the Early Barstovian. This curve was produced from a nested set of faunal lists (i.e., a type I curve of reference [10]), which started with one of the biogeographic provinces (Figure 1) and successively added species from the other ones, such that species accumulated as the sampling radius encompassed more and more of the western United States until all localities and provinces were included. Second, we examined unnested data, whereby species richness within each temporal bin for each of the nine different biogeographic regions was plotted against the geographic sampling area (type IV curve of reference [10]). Today, the continental biogeographic provinces we used are regarded as biogeographically distinct from one another [38–40], and it is likely that provincial differences also existed through the Oligocene and Miocene, although characteristics of each province were different from those of today [4,41].

Our metric for diversity is paleospecies richness, i.e., the maximum number of species identified in a sample. Because paleospecies richness is strongly dependent on many biases that influence sample size, we first standardized the data using rarefaction [42–46] based on occurrences (basically whether a taxon was present or absent at a given locality; see the Materials and Methods section for the alternative data parameters tested).

With the rarefaction results, we plotted the species richness values determined at 75 occurrences (a value that was high enough to see divergence in the rarefaction curves, but low enough to include many time intervals) against geographic area (the smallest polygon that enclosed all the localities contributing to a given species list). Analysis of the rarefaction curves and plots allowed us to estimate the impact of the species–area relationship on paleodiversity, and to distinguish peaks in diversity that are artifacts of the species–area effect from those that may indicate times of real diversity fluctuation.

## Results/Discussion

### Species–Area Effect with Nested Data

The species–area curve derived from nested data (Figure 2) within a single well-sampled time interval, the Early Barstovian (see Figure 1 and [34] for localities included), clearly demonstrates that species–area effects might underlie much of the diversity fluctuation apparent in paleontological datasets (Figure 2). We found that, just as for the modern species–area curves, the paleospecies–area curves for our data are described by a power function with a very high R^2^ value and a highly significant slope. It is not surprising that uncorrected species counts (Figure 2A) show a high R^2^ value (0.95) and significant slope (*p* < 0.0002), given the numerous biases in fossil data that are related to sample size. However, even after rarefying the data, a very strong species–area relationship (R^2^ = 0.85, *p* <0.0034) still existed (Figure 2B). This demonstrates that occurrence rarefaction by itself is insufficient to correct for size of geographic area, and that many of the apparent diversity fluctuations in paleontological datasets may be the result of the species–area relationship. This effect should be especially pronounced with the unequal geographic sampling that characterizes most paleontologic data.

**Figure 2:**
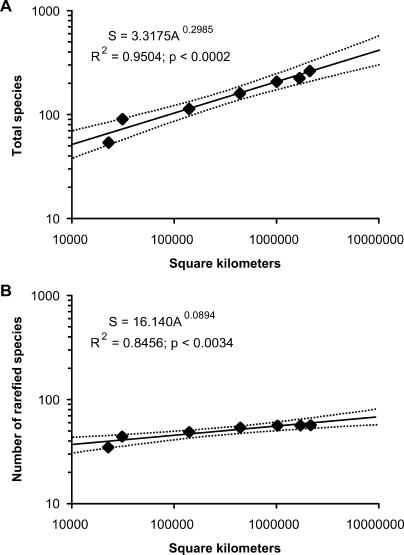
Paleospecies–Area Curve for the Early Barstovian Temporal Interval (A) Uncorrected counts of total species found in each successively larger geographic area. (B) Species richness values standardized by using the rarefaction value for 75 occurrences. Dotted lines are 95% confidence intervals around the best-fit line. Equation abbreviations: S, species; A, area.

### Species–Area Effect with Unnested Data

Plotting unnested data from each temporal bin for each of the nine biogeographic provinces (see Figure 1) further underscores how geographic coverage contributes to paleospecies diversity fluctuations (Figure 3). The paleospecies–area effect is highly significant (*p* < 0.001) in both uncorrected (Figure 3A) and occurrence-standardized assessments, but becomes a better fit with the standardized data (Figure 3B). This suggests that as other sampling biases are stripped away, the underlying control on paleospecies richness per time bin is geographic sampling area.

**Figure 3:**
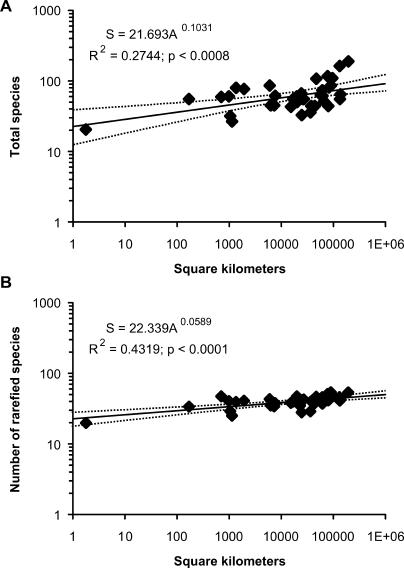
Number of Species Plotted against the Total Geographic Area of the Fossil Localities (A) Uncorrected counts of total species found in each temporal bin within each biogeographic province. (B) Species richness values standardized by using the rarefaction value for 75 occurrences. Each data point represents one biogeographic province in one of the temporal bins listed in Table 1. Dotted lines are 95% confidence intervals around the best-fit line.

### Paleodiversity Curves

Usually paleodiversity patterns are depicted as time series of sample-standardized data. In assessing apparent diversity change in such time series in different geographic regions (regions 1–3 and 6 in Figure 1), we found that the paleospecies–area effect by itself explains most of the apparent diversity fluctuations (Figure 4). The strength of the paleospecies–area effect becomes evident by comparing geographic sampling area through time with the “traditional” time-series depictions (Figure 4). In general, the peaks in the traditional time-series curves are species lists accumulated over large geographic areas, and the declines are species lists accumulated within smaller areas. This is clearly demonstrated by comparing paleodiversity fluctuations with geographic sampling area fluctuations (black lines versus gray lines in Figure 4). By determining the departures from this general concordance between geographic area and paleodiversity, it is possible to recognize which diversity fluctuations most likely have a cause rooted in biological process or sampling biases that are not predominantly caused by the species–area effect, as illustrated by the following examples.

**Figure 4:**
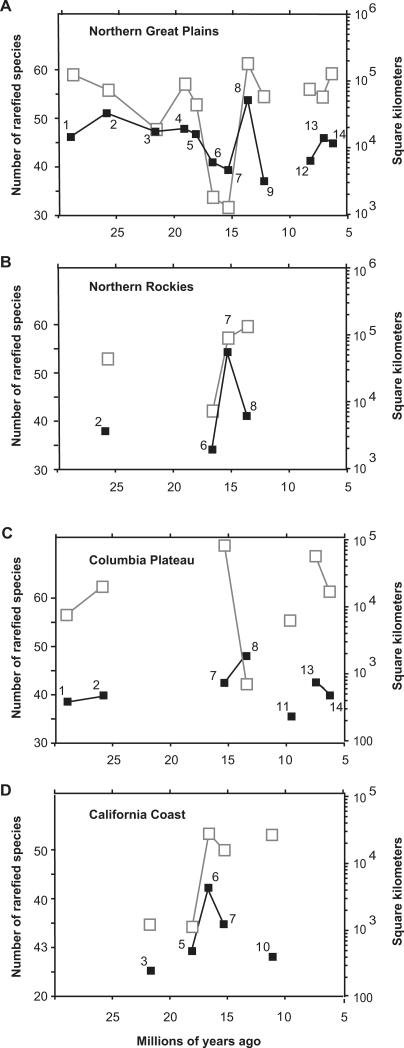
Comparison of Species Diversity and Geographic Sampling Area through Time Black lines and black boxes are time-series plots of occurrence-rarefaction species richness at 75 occurrences for each of the four biogeographic provinces. The numbers by the data points identify the time bins as indicated in Table 1; bin 1 is oldest and bin 14 is youngest. Gray lines and open boxes show the geographic area sampled for each time bin. (A) Northern Great Plains (region 3 in Figure 1). (B) Northern Rockies (region 2). (C) Columbia Plateau (region 1). (D) Coastal California (region 6).

In the Northern Great Plains (Figure 4A), the only fluctuations that are not predominantly explained by sampling differently sized geographic areas are in time bins 1 and 9 (lower diversity than expected) and possibly bin 13 (higher diversity than expected). The other fluctuations that would be interpreted from looking at the traditional time series alone (black line in Figure 4A), e.g., the dip and rise from time bins 4 through 8, are simply artifacts of the species–area effect. Likewise, in the Northern Rockies (Figure 4B), variable geographic sampling area explains most of the apparent time series fluctuations except low diversity in time bin 8; and in the California Coast (Figure 4D), only time bin 10 stands out as a true decline with respect to geographic area.

In the Columbia Plateau (Figure 4C), the time-series diversity fluctuations can generally be explained by the species–area effect, except for time bin 8, which exhibits a particularly pronounced deviation from that expected from geographic sampling area. In fact, that time features an unusual sampling situation, in which two geographically close faunas (Red Basin and Quartz Basin, Oregon) accumulated in two very different depositional environments [47], thus accounting for the rise in diversity even though geographic area decreased. In this case, the discrepancy between the diversity curve and the geographic area curve highlights an unusual sampling situation that must be taken into account when interpreting diversity fluctuations.

In addition, intraprovincial regression plots for these four regions were inspected to determine if they were consistent with the overall pattern seen in Figure 3. For the Northern Great Plains, Northern Rockies, and California Coast, the paleospecies–area regressions result in the expected positive slopes, but lack statistical significance likely because of the small number of points. For the Columbia Plateau, time bin 8 is such an outlier that it actually makes the slope slightly negative (though not statistically significant); however, with post hoc removal of time bin 8, the slope becomes significantly positive (R^2^ = 0.8524, *p* = 0.009), despite being based on only six remaining points. Like the time-series curves, such inspections are valuable in identifying time bins that may be recording unusual sampling biases, which are recognizable as extreme outliers.

Of additional relevance is the fact that the time series indicate that temporal coverage varies considerably from region to region. None of the regions have data for all of the temporal bins, and only the Northern Great Plains has a reasonably continuous time series. This suggests that paleospecies–area effects that we have demonstrated region by region may be magnified, rather than averaged out, by combining all geographic regions into a single dataset for time-series analysis. It will be enlightening to apply paleospecies–area corrections to the many paleodiversity curves that have been generated with other datasets as a prelude to interpreting the cause of apparent changes in diversity.

### Interpretation of *Z*-Values

*Z*-values are informative for species–area curves because they differ in datasets sampled from within provinces versus those that accumulate species from multiple provinces. Figure 2 exemplifies an interprovincial paleo-*z*-value. In general, the *z*-value for this Early Barstovian paleospecies–area curve is lower than for modern interprovincial species–area curves, as would be expected from the less complete sampling of the fossil biota as compared to the modern biota. The lower slope in the occurrence-standardized curve (Figure 2B) as compared to the uncorrected curve (Figure 2A) and to modern species–area curves also results from rarefaction of the localities [48]. The *z*-values for regressions illustrated in Figure 3 are for unnested data and thus are not directly comparable to *z*-values for type I species–area curves. However, it is worth noting that their *z*-values also are low compared to either inter- or intraprovincial species–area curves [8], as would be expected from the rarefaction effect.

### Conclusions

Correcting the species–area bias in paleontological data is different from the indirect proxies for sample standardization that have been applied in the past, such as standardizing for rock volume or outcrop area, because it actually takes into account dispersion of sampling sites, rather than area potentially available for sampling. Our demonstration that the paleospecies–area relationship explains many of the spatiotemporal differences that have been traditionally seen in species richness of Miocene mammals has widespread implications.

First, it is likely that the influence of the paleospecies–area relationship is similarly strong in most fossil datasets, meaning that the shapes of global and regional paleodiversity curves may change substantially once the area effect is accounted for. This indicates that it may now be possible to refine our understanding of widely recognized events in the history of life by correcting for the paleospecies–area effect. For example, taking into account paleospecies–area effects will be important in clarifying the controversy about whether marine diversity substantially increased in the Cenozoic—the so-called pull of the recent [5,49]. Paleospecies–area corrections can be expected to adjust magnitudes and perhaps timing of extinction events documented in the fossil record, which in turn are of particular value in comparisons of past with current extinction rates [9,25,50,51]. Interpretations of ecological dynamics through time [6,30,35,49,52], the cornerstone of inferring macroecological processes from pattern [11,53], also are subject to paleospecies–area considerations.

Second, it has been prohibitively difficult to correct for area effects on paleospecies richness, but with interactive mapping and image analysis software applied to paleontological databases as we have done here, such corrections are now feasible. Finally, the recognition that paleospecies–area curves exhibit some of the same properties (high R^2^ values, significant slopes) as modern species–area curves portends an important way to merge macroecology with paleontology.

## Materials and Methods

### Temporal bins

We followed published literature in assigning fossil occurrences to subdivisions of the North American Land Mammal Ages as specified in Tedford et al. [41]. Although the temporal bins are not equal in duration, we determined that this has little influence on diversity counts per bin because (i) there is no correlation between bin length and number of localities (R^2^ = 0.04); (ii) the localities do not span the entire time represented by each bin [34]; and (iii) recent work [54] has indicated that bins of the sort used here, based on maximum taxon associations, are best suited to comparisons of diversity through time, as they produce a series of biologically meaningful groupings that do not change much within each bin.

### Species counts

We calculated both maximum and minimum number of species, the former including all specimens that were only identified to genus or higher taxon as belonging to unique species, the latter including all such taxa as belonging to a species represented by more diagnostic material. We present the minimum counts here, but using maximum counts does not substantively alter our results.

### Geographic area calculation

Geographic areas were calculated by using the MIOMAP mapping interface to zoom in on the set of localities at appropriate scales, importing the resulting image into the image analysis program ImageJ (http://rsb.info.nih.gov/ij/, and then using the ImageJ software to trace the minimum convex polygon that would enclose all the localities of interest and to calculate the area enclosed by the polygon. For our purposes this was the most appropriate measure of geographic area (rather than summing radii around localities or smaller polygons that minimally enclosed spatially discrete clusters of localities, but not counting the area that separates the clusters) because the intent was to see how species accumulate as total geographic area is added.

### Rarefaction methods

Because paleospecies richness is strongly dependent on biases that influence sample size, we standardized the species counts using rarefaction [42–46]. Rarefaction was accomplished with S. Holland's analytic rarefaction software (http://www.uga.edu/strata/software/, based on the analytical solutions to rarefaction presented by Raup [42] and originally derived by Hurlbert [44] and Heck et al. [45]. A review of the development of this methodology can be found in Tipper [43]. Detailed study of the taphonomic and collection biases of all included fossil sites can identify whether rarefaction using number of identified specimens (NISP—how many specimens represented a given taxon) or occurrences (whether a taxon was present or absent at a given locality) is more appropriate; however, that is usually prohibitively labor-intensive. Because either method might be appropriate in a given instance, we analyzed our data in both ways to understand the range of results that might be produced. We found that many of the NISP counts are based only on “high-graded” specimen counts—i.e., publication of only the best specimens, rather than all the specimens that were collected from a given locality [55]. Time intervals that have many of these high-graded NISP counts, combined with many localities that have very poor NISP information, exhibit a rarefaction curve that rises artificially steeply. Rarifying by occurrences removes the effect of high-graded localities and missing data. In addition, this method produces results equivalent to another commonly used method of sample standardization—occurrences weighted resampling [46]. Given the benefits of employing occurrence rarefaction, we report only those results here. However, it is worth noting that using the NISP rarefactions would not alter our conclusions. We determined species richness at 50, 75, and 100 occurrences. We report only the richness values at 75 occurrences, because that occurrence value provided a large number of data points while at the same time eliminating points that were based on more suspect, spotty data. Interpreting results on the bases of 50 or 100 occurrences would result in substantially similar conclusions.

## Abbreviations

MIOMAP: Miocene Mammal Mapping Project
NISP: number of identified specimens
